# Generative AI as a transformational logic for cognitive neuroscience

**DOI:** 10.1038/s42003-026-10642-w

**Published:** 2026-07-08

**Authors:** Christian Beste, Shervin Safavi

**Affiliations:** 1 Cognitive Neurophysiology, Department of Child and Adolescent Psychiatry, Faculty of Medicine, TU Dresden, Dresden, Germany; 2 German Center for Child and Adolescent Health (DZKJ), Partner Site Leipzig/Dresden, Dresden, Germany

**Keywords:** Cognitive neuroscience, Computational neuroscience, Psychology

## Abstract

Cognitive neuroscience faces a paradox: neural data are abundant, yet conceptual synthesis has stalled because dominant contrast-based approaches show where activity differs but not how cognitive operations relate or transform. Here, we propose a generative-transformational logic grounded in AI and neural geometry, treating cognition as lawful mappings among neural states. Generative models can learn latent transformations linking states across tasks, contexts, and individuals. Because transformation success is testable, this framework enables counterfactual simulation and connects data-driven modeling with theory-driven inference. It moves cognitive neuroscience from mapping correlates toward algorithmic explanations of how the brain generates and reorganizes cognition over time.

## Abstract (web summary)

This Perspective discusses how generative AI can help cognitive neuroscience move beyond contrasts by modeling transformations among neural states and linking neural data, counterfactual simulation, and theory.

## From neural data to cognitive organization

Over the past few decades, cognitive neuroscience has accumulated abundant high-dimensional data, including intracranial recordings in humans and animals, EEG and MEG recordings, and high-resolution fMRI. Through open science^1^, large-scale repositories and research initiatives, scientists have access to such data to an extent not seen before^2–7^. Yet, central questions in the field of how cognitive operations are organized, interrelated, and transformed within the brain’s neural architecture remain unresolved. We can increasingly specify where and when neural activity changes and may shape the cognitive processes that give rise to behavior^8^. However, we still lack clarity about how nominally different cognitive operations and underlying neural processes relate to one another.

## The case for an additional logic of inquiry

Cognitive neuroscience faces a conceptual rather than a data bottleneck, necessitating a reconsideration of the logic underlying its use of data to draw inferences about cognitive brain functions. Once data has been collected, researchers face the challenge of determining how to extract mechanistic insight from high-dimensional signals that resist intuitive interpretation and overwhelm traditional analytic frameworks. Paradoxically, as data accumulation has outpaced conceptual synthesis, it can now provide momentum to the field by offering a basis for a shift in research logic enabled by generative artificial intelligence (AI) that can also be of relevance for technical implementations^9^.

Cognitive neuroscience has been built on a subtractive logic. Through subtractive logic, one infers cognitive processes by comparing conditions to identify differences in neural or behavioral signals. Experimental conditions are subtracted from each other to identify differences in neural activity or behavioral performance^10–13^. This approach has yielded robust insights into the neural underpinnings of cognitive functions; however, it cannot determine whether the same neural mechanism underlies two cognitive operations. Even when activity patterns overlap, such overlaps can reflect coincidental co-activation rather than shared computation^14–16^. It is increasingly evident that a single neural process can support multiple cognitive operations. This flexibility arises, for instance, from neural reuse and mixed selectivity, enabling the same neuronal populations to combine task variables in different ways. For example, sensory-parietal neurons can encode both current and mnemonic cues, linking perception, memory, and decision-making within a shared population code^17^. Likewise, cognitive flexibility may reflect the dynamic reuse of fronto-parietal control mechanisms across tasks^18–20^. Mixed-selectivity architectures^21^ and dynamical systems accounts^22,23^ show how nonlinear input combinations expand representational dimensionality, allowing diverse functions to emerge from one neural substrate. Thus, a single neural process can represent multiple cognitive operations, reflecting the efficiency and compositional flexibility of the brain’s computational architecture. Contrasts are indispensable for identifying where cognitive operations diverge. Yet, they fail to capture the relational architecture that governs how cognitive states transform into one another, as suggested by neural reuse or mixed selectivity principles. Without such transformability, the brain would fragment into isolated modules, which is unlikely the case^24–27^. This is also discussed in the context of shared domain-general mechanisms or distinct domain-specific systems across various fields of cognitive neuroscience^28–36^.

The concept of the brain reusing neural systems for different purposes is also evolutionarily justified because it represents a mechanism for efficiency and compositional flexibility. Instead of developing entirely new, specialized neural circuits for every novel cognitive task, evolution favors the repurposing of existing, robust neural substrates (i.e., neural reuse)^37^. This allows expanding the brain’s representational capacity without requiring a massive increase in anatomical wiring, leading to a compact and highly adaptable computational system that is more energy-efficient and better equipped to handle a dynamic environment^14^.

Thus, transformational logic, rather than subtractive logic, is indispensable for asking how different cognitive operations and their neural underpinnings are related. A transformational logic infers cognitive processes by modeling the transformation that links states, using generative models to reveal latent structure and mechanisms. Here, generative modeling becomes a tool for testing claims about shared versus distinct mechanisms underlying different control operations. While architectures that translate one state into another could be interpreted purely as conditional mappings, they are utilized here as generative models because they learn to synthesize high-dimensional data samples by sampling from learned data distributions, rather than performing deterministic, point-to-point transformations.

**Fig. 1:**
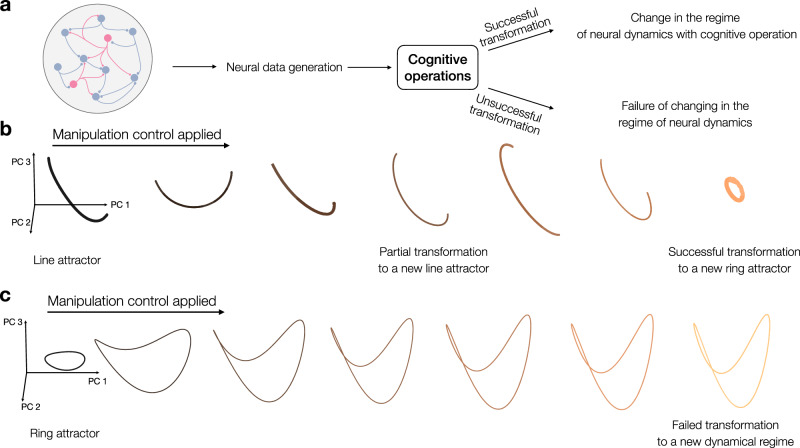
Examples of two neural manifolds under manipulations. **a** Schematic depiction of successful and unsuccessful transformation under cognitive operation (see **b**, **c** for more details). The left panel depicts a schematic of a neural network consisting of several neurons that are recurrently connected, thereby can generate diverse forms of network dynamics. **b** Neural manifold of a network implementing a ring attractor (in the coordinates of the top three principal components of the network dynamics) that does not change (remain a ring attractor) the neural manifold under manipulation (an operation on the manifold). The intensity of the color indicates the deviation of the ring from a planar structure, and becomes increasingly warped in three-dimensional space. **c** Similar to (**a**), the neural manifold is visualized in the top three principal components of the network activity, where increasing color intensity indicates the gradual collapses of a ring attractor into a line attractor, reflecting a complete transformation. First example (**a**) depicts a case of neural dynamics that remains unchanged, even under manipulations; however, second example (**b**) indicates an example where neural dynamics transform to a distinct regime of dynamics under manipulations. This can be a complete transformation (from a line attractor to a ring attractor; see two extremes of **b**), or remain a line attractor (failed transformation) or an intermediate transformation (sixth manifold of **b**), which is an intermediate manifold between a ring and a line attractor. The neural network schematic in panel a was adopted from the open-source scidraw.io repository.

To better illustrate our approach, we use the notion of operation on attractor dynamics that have been suggested to serve as a potential substrate of cognitive computations: a ring attractor in the context navigation and a line attractor for working memory (see Fig. 1a)^38^. Attractors are neural networks whose dynamics elicit activity, and they reside in a characteristic low-dimensional space. In computational neuroscience, continuous attractor networks provide a fundamental mechanism for how the brain maintains stable representations of information over time. Ring attractors are characterized by a circular manifold of stable neural states. They are important in spatial navigation, where a continuous localized bump of neural activity moves smoothly along the ring to dynamically track periodic variables, such as an animal’s heading direction. In contrast, line attractors allow neural activity to stabilize at any point along a linear continuum. This property makes them crucial for working memory, as they enable neural circuits to persistently hold and maintain graded, non-periodic continuous variables (such as specific quantities, spatial locations, or eye positions) long after the initial sensory stimulus has been removed. These attractors may or may not transform under manipulation (depending on the nature of the manipulations; see Fig. 1b, c). These manipulations can cover a diverse range of changes in the network, for instance, deactivation of neurons due to inhibition and increase in excitability of neurons due to external input, that all can lead to a change in the resulting neural manifold. Successful transformation of neural processes (Fig. 1a) can imply that the two states or cognitive functions share a common ground (see Fig. 1b), where neural dynamics transform to a distinct regime of dynamics under manipulations (here, from a line attractor to a ring attractor). This transformation can also be partial (Fig. 1b, partial transformation). Furthermore, there can be a failure in the transformation of these states that implies functional separability (see Fig. 1c, in which neural dynamics remain unchanged even under manipulations). If overlapping neural activity merely reflects two independent, non-interacting processes occurring in the same anatomical region, a generative model trained on the dynamics of one state might fail to accurately predict or transition into the other, because their underlying representational geometries and transition rules might fundamentally differ. By contrast, if a generative model can systematically transform the neural dynamics of one cognitive operation into another, it demonstrates that the two states are likely linked by a shared set of computational rules. This allows us moving the level of analysis from static spatial overlap to dynamic, algorithmic dependence.

Furthermore, a transformational approach is also necessary, considering that cognitive functions are not categorical but continuous, relational, and context-dependent^39^: regarding continuity, cognitive states rarely exist as discrete, all-or-none entities, and the same also applies to cognitive dysfunctions, given comorbidities and transdiagnostic dimensions in psychiatric conditions. Processes such as attention, control, or memory load evolve gradually along multiple dimensions (e.g., intensity, uncertainty, and relevance), and their neural correlates vary as well^40^. Treating these continuous phenomena as binary conditions, for example, conflict versus no conflict, is often experimentally useful because it maximizes condition differences, improves statistical power, and reduces potential confounds. Such discretization is also unavoidable to some degree, because empirical designs always sample a finite number of conditions from a potentially continuous space. Our point is therefore not that binary contrasts are inappropriate, but that they answer a restricted class of questions: they can establish whether two sampled endpoints differ, but they provide limited information about how intermediate states are organized, whether transitions are smooth or nonlinear, and whether endpoint differences reflect a shared trajectory or separable state spaces. Regarding relationships, cognitive operations derive their identity not in isolation but from their relations to other states, as, for example, shown for attention and working memory processes^41^. Studying cognition thus requires examining the geometry of relations among states rather than treating processes as independent modules. Concerning context dependency, the same neural pattern can serve different functions across contexts, and different patterns can implement similar functions depending on goals, expectations, or motivational states.

Conventional statistical analyses, however, impose categorical contrasts that discretize otherwise continuous neural and cognitive dynamics (e.g., congruent vs. incongruent, correct vs. incorrect, or patients vs. controls). This forced discretization imposes artificial boundaries on processes (i.e., conflict, control adjustments, error likelihood, affective intensity, or symptom severity) that are inherently continuous and graded. As a result, the rich information of neural and cognitive dynamics is reduced to a set of binary contrasts, which can obscure transitional states and discard variance that may carry explanatory value^42^. Transformational analyses, in contrast, preserve the geometry of these relations, enabling researchers to trace how one cognitive operation may gradually transform into another rather than merely establishing that they differ. A related descriptive perspective comes from large-scale functional connectivity gradient approaches in systems neuroscience^43–45^. These approaches use similarities in regional functional-connectivity profiles to arrange cortical regions along continuous, low-dimensional axes, rather than assigning them only to discrete networks^45^. For example, the principal cortical gradient describes a macroscale axis from sensory/motor systems to transmodal/default-mode regions^43^, and related work has framed such gradients as intrinsic coordinate systems of cortical organization^44^. Such gradients are best understood neither as the generative process itself nor as a complete account of cognitive transformation, but as empirically derived coordinates of a latent organizational space. They summarize dominant axes along which neural systems are arranged and can therefore provide a coordinate system within which task-related dynamics or transformations may be analyzed^43,44^. From this perspective, gradients can be viewed as emergent coupling structures, whereas generative models aim to explain how such large-scale organization is produced, perturbed, and transformed across tasks, contexts, and individuals.

In other words, these simulations allow testing mechanistic hypotheses about how such large-scale organization and its constituent dimensions are dynamically organized or reorganize in reaction to a perturbation. This rationale has already been key to motivating a geometric approach to brain data^46^. Recent years have seen a shift from statistical to geometric approaches in neuroscience^12^, emphasizing how neural population activity forms structured manifolds^47,48^ in high-dimensional representational spaces^49–53^. These approaches have revealed how cognition is reflected in the geometry of neural states (i.e., their distances, curvatures, and trajectories). In single-unit and multi-unit recordings, distances in latent space have been used to quantify how similar the prefrontal cortex encodes different task rules or action goals^54,55^, while curvature along neural trajectories can reveal how representations transition from stimulus evaluation to action selection^23^. In fMRI, manifold orientation has been shown to rotate across task epochs (e.g., from encoding to maintenance to retrieval in working memory tasks), indicating dynamic reconfiguration of cortical coding schemes^56^. In EEG/MEG, geometric analyses capture how trajectories of oscillatory activity diverge for different control demands^57,58^. This geometry of neural states stance stems from long-standing attractor-network and dynamical-systems models in computational neuroscience, which describe cognition as trajectories through a high-dimensional energy landscape^23,59^. Critically, however, the mapping of relationships among observed states in these approaches cannot explain how such states emerge or generalize to other states. Being able to do so is essential because this would allow researchers to test counterfactuals (e.g., how neural processes would respond under altered control demands) and to infer the latent mechanisms shaping cognition. Counterfactual reasoning (What if neural state X were perturbed?) is the hallmark of a mechanistic approach^60^ that provides us a rule or generative process that explains how cognitive states possibly evolve and relate to one another^61^.

## Generative AI can enable an additional logic of inquiry

Generative AI, such as conditional generative adversarial networks (GANs), variational autoencoders (VAEs)^62,63^, or latent diffusion models (LDMs)^64,65^, offers a powerful and complementary perspective to current geometric approaches by shifting focus from the structure of representational spaces to the processes that generate and transform them. While geometric approaches to brain function delineate how neural states are arranged (for example, similarity of neural activity across different behavioral epochs, e.g., stimulus vs. decision), generative AI models estimate and learn the latent rules that can possibly produce and reshape this geometry^66,67^ (generating realistic neural activity based on behavioral context). In standard GANs, a generator maps a latent noise vector to a realistic data distribution. However, to model transitions between cognitive states, our proposed framework specifically relies on cycle generative adversarial networks (cGANs). In a standard conditional generative adversarial network, the generator samples from a latent variable while being conditioned on additional information, such as a task label, cognitive state, or behavioral context. For transformational analyses of neural data, the more relevant class of models is conditional domain-translation architectures. In such models, the generator can be conditioned on empirical neural activity from a source state, for example, a low-conflict trial, and trained to synthesize samples matching the target-state distribution, for example, high-conflict trials. If source and target observations are paired, pix2pix-like conditional adversarial translation may be appropriate; if they are unpaired, cycle-consistent domain translation may be more suitable. When no effective stochastic component is used, the model should be interpreted as a deterministic or approximately deterministic neural-state translator rather than as a full probabilistic generator.

Variational autoencoders (VAEs), in turn, learn an explicit probabilistic mapping between latent (cognitive state dynamics) and observed spaces (measured neural activity), allowing the model to learn a latent low-dimensional representation of the empirical data that is often easier to interpret. Variational autoencoders, as autoencoders, achieve this by first compressing high-dimensional observed data (e.g., multi-channel EEG or fMRI voxel patterns) into a low-dimensional probabilistic bottleneck (the encoder), and then reconstructing the original neural activity from this compressed representation (the decoder). However, using variational autoencoders (in contrast to autoencoders) yield to a latent manifold that remains continuous and smooth. Variational autoencoders enforce a probabilistic constraint (typically a Gaussian prior) on the latent representations. This regularization forces the latent manifold to remain continuous and smooth. This enforced smoothness is critical for the transformational logic discussed here because every point along the variational autoencoders’ latent manifold corresponds to a potential coherent neural state. Therefore, it enables the valid interpolation required to model graded, continuous transitions between distinct cognitive states and allows for the simulation of plausible counterfactual data. Furthermore, by forcing the network to auto-encode the measured neural activity with minimal information loss, the resulting latent space is structured to potentially capture low-dimensional patterns within the observed data. However, the outcome and suitability of the low-dimensional representation depend on the nature of the data. Within our proposed framework, we conceptualize these latent dynamics as the underlying cognitive state dynamics (or the subspace that cognitive processes may exploit to operate).

Thus, the variational autoencoder provides a potential bridge: the encoder maps high-dimensional neural observations to putative cognitive states, and the decoder maps those states back to testable neural predictions. However, it is crucial to note that variational autoencoders do not yield interpretable latent dimensions by default (see the section on “Caveats of using generative AI for cognitive neuroscience”). Alongside generative adversarial neural networks and variational autoencoders, LDMs offer a conceptually distinct and highly relevant approach for cognitive neuroscience. Rather than learning a direct mapping, LDMs generate data via stochastic, iterative trajectories through a latent space, progressively denoising representations to uncover structure. For example, let us consider the process underlying decision and action selection. The underlying cognitive process for action selection (latent space) remains the same irrespective of observation (the measurement modality, M/EEG, MRI). Therefore, the key is learning the mapping from the latent process to neural observations, which we believe generative AI can greatly facilitate. Furthermore, for some models, such as LDMs, the generation process might also align with dynamical and geometric interpretations of neural activity. Because they explicitly model the gradual evolution of states over time (e.g., in a form similar to evidence accumulation that has been formalized in drift diffusion models of decision making). Together, these models not only describe representational geometry but also potentially the ways the underlying neural processes emerge and change.

Generative modeling is increasingly recognized in cognitive neuroscience^51,68–72^. These approaches treat neural data not merely as isolated points, but as residing on transformable manifolds, low-dimensional geometric structures embedded within the high-dimensional measurement space (e.g., sensor or voxel space) that capture the coherent states, neural system can occupy (for example, observation of default-mode network in neuroimaging data suggests brain regions operate in coordination). Thus, a valid transformation between two cognitive operations may operate in low-dimensional subspaces capturing these correlation structures. Generative modeling can broaden the methodological and conceptual repertoire of cognitive neuroscience by shifting the focus from representational geometry to the processes that generate and transform cognitive states. An example of how such an AI-based framework of cognitive neuroscience research can be implemented is provided in Box 1.

Importantly, the transformational criterion proposed here is not intended as a direct claim about biological implementation. A generative model implemented in silicon does not become a mechanistic model of the brain merely because it can map one neural data distribution onto another. Its relevance lies instead in its role as a constrained formal probe: it tests whether empirically measured neural states can be related through a transformation that preserves neural geometry, task-relevant information, and behavioral predictability. Biological interpretation requires additional validation, including held-out prediction, comparison against simpler statistical mappings, perturbational or causal tests where possible, and convergence with neurophysiological knowledge. Thus, transformation success should be interpreted as evidence that a shared representational description is viable, not as proof that the model’s internal operations are biologically implemented.

Box 1: an example AI framework To illustrate how the proposed framework can be implemented in practice, consider a hypothetical but realistic EEG dataset in which participants perform a parametrically varying conflict-monitoring task (e.g., a continuous Flanker or Stroop paradigm). The dataset comprises trial-wise EEG time-frequency decompositions, single-trial behavioral indices (reaction time and accuracy), and metadata regarding trial context (conflict level, expectancy, and previous-trial history).

*Step* 1 (*formulate relational hypotheses*): instead of contrasting high vs. low conflict trials, one can pose the relational question of whether a generative model learns to transform the neural signatures of low-conflict trials into those observed under high conflict? If successful under appropriate validation, such a transformation would be consistent with the hypothesis that low- and high-conflict trials occupy a shared representational state space for control demands. However, this would not by itself demonstrate that the brain implements the same transformation as the artificial model. Conversely, transformation failure would not prove distinct biological mechanisms, but would indicate that, under the tested model class, data modality, and task constraints, the two states are not captured by a common transformation.

*Step* 2* (dataset identification)***:** the dataset provides continuous neural recordings aligned to behavioral outputs and context variables. Preprocessing yields single-trial spectral maps (theta/alpha/beta power, connectivity estimates). These structured, high-dimensional representations are suitable for training conditional generative or domain-translation models. Depending on the data structure, this may involve a stochastic conditional generator, a pix2pix-like paired translation model, a cycle generative adversarial neural network, like unpaired domain-translation model, a conditional variational autoencoder, or a conditional diffusion model.

*Step* 3 *(quantify representational geometry)*: a variational autoencoder is trained to embed all trials in a latent space. When multiple conflict levels or trial-wise continuous conflict estimates are available, latent trajectories can reveal whether conflict varies along a single continuous axis (e.g., frontal theta scaling), follows a nonlinear trajectory, or separates into distinct regions of state space (e.g., beta suppression, cross-frequency coupling).

*Step* 4 *(estimate transformation and computational principles)*: one then trains a conditional domain-translation model that maps latent embeddings or neural features from low-conflict conditions toward the distribution observed under high conflict. If the goal is to model trial-by-trial variability, the generator should include an explicit stochastic component, for example, a latent noise vector, a sampled variational autoencoder latent state, dropout used at inference, or a diffusion process. This distinguishes a stochastic conditional generator from a deterministic feedforward network. A deterministic translator can learn a useful point-to-point mapping, but it does not by itself model the variability of possible target-state trajectories. By contrast, a stochastic conditional generator can be evaluated by repeatedly sampling outputs for the same source state and testing whether the resulting distribution matches the empirical target distribution in terms of reconstruction fidelity, representational geometry, and functional equivalence. By sampling from this distribution, the generative model captures the probabilistic, one-to-many nature of neural transitions, generating diverse, biologically realistic single-trial trajectories that may reflect how cognitive operations are realized.

*Step* 5 *(generalize across tasks, domains, individuals)*: once trained, the model can be tested on (i) other conflict tasks (e.g., Simon task), (ii) other populations (e.g., attention deficit hyperactivity disorder, depression, aging), and (iii) using different sources of neural data (MEG, intracranial EEG). Success would suggest that conflict-related neural geometry is shared across domains, consistent with a common low-dimensional control manifold. Here, the control manifold refers not simply to a geometric correlate, but to a representational state space whose structure supports similar control-related transformations across tasks. Its functional relevance would therefore be established only if it enables transfer—such that mappings learned in one domain successfully predict neural or behavioral responses in another.

*Step* 6 *(Integrate into theory building)*: the results from Step 5 have direct implications for the understanding of the neural basis and the computational basis of the related cognitive concepts. For example, if neural data from a visual cognitive control task can be successfully generated from auditory control dynamics, this computationally constrains the underlying theory to rely on a modality-general, shared algorithmic workspace. Conversely, if generative transformations fail between two nominally similar tasks, this would not by itself prove that their biological algorithms are orthogonal or modular. Rather, it would constrain theory by suggesting that the measured neural state spaces are not related by the tested transformation. Such a result would motivate further tests of whether the failure reflects genuinely separable computational structure, insufficient model capacity, inappropriate inductive biases, measurement limitations, or unmodeled task variables. These levels are directly related to each other, or become better related through the identified transformative principles on a mathematical level. Importantly, this does not displace traditional neural, computational, and cognitive levels of explanation. Rather, it reframes their relation: generative models act as bridges across levels by learning transformations that connect neural data, latent computational structure, and cognitively meaningful state changes. Their contribution is therefore not to privilege one explanatory level, but to test whether hypotheses formulated at one level can generate coherent predictions at another. In this way, generative modeling supports integration across levels rather than replacement of existing explanatory frameworks.

*Step* 7 *(closed-loop integration):* based on Step 6, the success or failure of these generative mappings refines theoretical claims: if transformations succeed across tasks, a domain-general control mechanism is supported. If transformations fail across tasks but succeed within tasks, a task-specific control geometry is implied. If patient data cannot be generated from healthy manifolds, pathological distortions of representational geometry are inferred. Through this process, new questions may arise that provide the basis of a new Step 1 to repeat this framework.

## Proof-of-principle applications

Binz and colleagues^68^ provide one of the clearest demonstrations of this shift. Using state-of-the-art large language models (LLMs), they show that latent representations learned from cognitive task data can be systematically perturbed to emulate the transformation from one task demand to another. By manipulating generative factors, they test whether different cognitive regimes occupy a shared manifold or require distinct latent transition rules. This introduces generative manipulations (i.e., What would a high-conflict trial look like if generated from low-conflict input?) as empirical probes into the organization and continuity of cognitive state spaces. However, the role of transformer-based architectures, such as the LLMs used in these paradigms, requires careful qualification in the context of transformational logic. While transformers excel at learning complex mappings over symbolic or sequential spaces, they do not inherently impose the continuous latent geometry necessary for mapping gradual cognitive transformations. Unless explicitly designed or constrained to do so. Their representations may not naturally yield the smooth, differentiable manifolds required to model continuous neural and cognitive transitions. Thus, while they are promising tools for sequential behavioral prediction, their utility for geometrically mapping intermediate cognitive states must be evaluated critically.

Another example demonstrating the fidelity of generative modeling for cognitive neuroscience was provided by Cheng et al.^69^ applying this logic in a multimodal context. Their work demonstrates that generative models can learn a shared latent process that gives rise to fluctuations in EEG, fMRI signals, and behavior. Instead of correlating signals across modalities, their approach identifies the generative mechanism that produces them, hence allowing explicit tests of whether different measurement spaces reflect the same underlying cognitive variable. Crucially, misalignment or divergence in the learned latent space reveals where modalities encode distinct aspects of cognition, offering a principled way to evaluate theories of integration, dissociation, or processing hierarchy. Another example comes from Ji-An and colleagues^70^, who show that small recurrent neural networks (RNNs) can outperform classical cognitive models in predicting human and animal decisions. Importantly, these tiny RNNs can be interpreted using tools from dynamical systems theory, translating the learned policy into a vector field that describes how preferences, beliefs, or internal states evolve. Tiny RNNs can be used to decode a kind of black box of these principles and explain performance. These networks thus serve as empirical generators of cognitive algorithms, recovering latent dynamical rules rather than merely encoding representational structure (see also^73^ ref., for a comprehensive review on broad application of RNNs for recovering underlying computational rules). Finally, Vahid et al.^72^ use conditional generative adversarial neural networks ask whether the neural dynamics of one cognitive process (response inhibition) can be generated from those of its antagonistic counterpart (speeded response execution). Using EEG data, they show that inhibitory-control signals can be accurately generated from go-trial activity. Where the transformation fails, the model reveals subprocesses that cannot be inferred from execution dynamics alone. Here, generative modeling becomes a tool for testing claims about shared vs. distinct mechanisms underlying antagonistic control operations^74–76^.

These studies outline the potential applications of AI and how neural data are used to infer cognitive architecture. Generative AI will enable counterfactual simulation. Ultimately, through generative transformations, one can simulate how neural trajectories would evolve if attention were reallocated earlier, if inhibitory demands were absent, or if internal variables, such as uncertainty or reward expectation, were selectively altered. Likewise, models can generate mappings between perceptual and imagined states or simulate how neural trajectories in clinical or developmental populations might unfold under normative control dynamics. In this way, generative AI ideally could serve as a virtual laboratory in which mechanistic hypotheses about cognitive processes can be instantiated and explored before being subjected to empirical testing. From this, two complementary forms of counterfactual inquiry emerge: conceptual counterfactuals probe a model’s internal dynamics under hypothetical perturbations, revealing which transformations are lawful or possible within the trained models. Empirical counterfactuals, in contrast, apply these learned mappings to new or unseen neural data to predict how real systems would respond under corresponding manipulations. Together, these approaches enable in silico simulation of experimental outcomes, providing a powerful means to forecast how neural and behavioral patterns would change under controlled transformations. Integrating this generative logic with empirical validation could bring enormous predictive capacity and possibilities for in silico intervention. Methodologically, generative modeling unites data-driven modeling with theory-driven inference by learning how latent causes give rise to observable data. This allows empirical regularities to constrain theoretical assumptions while maintaining the model’s mechanistic interpretability. The success or failure of a generative transformation becomes a quantifiable test of representational overlap or segregation—allowing theoretical constructs, such as shared mechanism or domain specificity^29–32,34,35^ to be evaluated.

## Interpretability and analytical access to learned transformations

Crucially, generative AI models can not only demonstrate whether neural states are transformable but also expose the mathematical principles underlying such transformations^66,72,77,78^. Interpretability methods now make these principles tractable (see Table 1).

**Table 1:** Analytic approaches in generative model–neuroscience integration

Analytic approach	Core idea	Tools/methods	What it reveals	Example/interpretation
Latent manifold analysis	Embed neural data and generated reconstructions in the same latent space to study internal geometry.	Principal component analysis (PCA), manifold alignment, geodesic distance measures.	Organization, curvature, and clustering of latent trajectories between cognitive conditions.	Shows how one cognitive state morphs into another through continuous trajectories in latent space.
Jacobian and sensitivity analyses	Use the local Jacobian of the generator to quantify how small input perturbations change the output.	Derivative mapping, gradient-based sensitivity measures.	Identifies causally influential neural features driving transformations.	Reveals dependencies, such as how frontal-theta fluctuations modulate parietal-alpha under increased control demands.
Representational alignment across layers	Compare internal layers of the model to cortical processing levels.	Representational dissimilarity matrices (RDMs), layer-to-brain alignment analysis.	Maps correspondence between model stages and neural hierarchies.	Early layers → sensory features; intermediate → sensorimotor integration; deep → abstract/goal-related codes.
Information-theoretic decomposition	Quantify which latent dimensions carry explanatory power for cognitive transitions.	Mutual information, total correlation, and entropy-based metrics.	Identifies which neural features are associated with specific latent factors and tracks information redistribution.	Links features, such as phase synchrony or cross-frequency coupling to latent dimensions governing transformation.

These can include analysis of latent dynamics through manifold analysis with tools such as principal-component analysis, manifold alignment, or geodesic distance measures in the model’s latent space^79,80^. Notably, neural manifold learning methods are rapidly growing in neuroscience in diverse directions. While conventional linear manifold learning methods (e.g., principal component analysis) have been widely used and have been insightful, recent nonlinear manifold learning methods offer even more potential as they allow us to learn topological features that are central to understanding cognitive processes. Furthermore, manifold analysis can also refer to Jacobian and sensitivity analyses, which quantify how small perturbations in input features affect the output representation. This can reveal conditional and potentially nonlinear dependencies that go beyond simple correlations in band-limited activity. For example, sensitivity or Jacobian analyses can quantify how perturbations in features related to frontal theta alter the model’s transition toward neural states associated with higher control demands, and whether this occurs together with systematic changes in parietal alpha. In this way, the model does not merely identify co-fluctuation between bands but generates testable hypotheses about how specific features contribute to state transformations within a learned latent dynamic.

Moreover, representational alignment across neural data and neural networks performing the same task is also a promising direction. For instance, by comparing representational dissimilarity matrices of layers of deep neural networks with those derived from neural data recorded from different stages of the processing hierarchy, one can identify which stages of the model correspond to which levels of cortical processing^81,82^. However, such similarity does not necessarily imply similar computations (see^83–85^ for other complementary perspectives). Finally, information-theoretic decomposition may be useful since its measures can quantify which latent dimensions carry the most explanatory power for a given cognitive transition^86,87^. These metrics enable the identification of which neural features (e.g., phase synchrony, connectivity motifs, or cross-frequency coupling) are systematically associated with specific latent factors, and how task-relevant information captured by a given recording modality is re-expressed across regions during transformation. Importantly, such analyses provide access only to modality- and model-dependent aspects of neural information, not to the full information content available to the brain (see Table 1).

## Theoretical implications for cognitive neuroscience

**Fig. 2:**
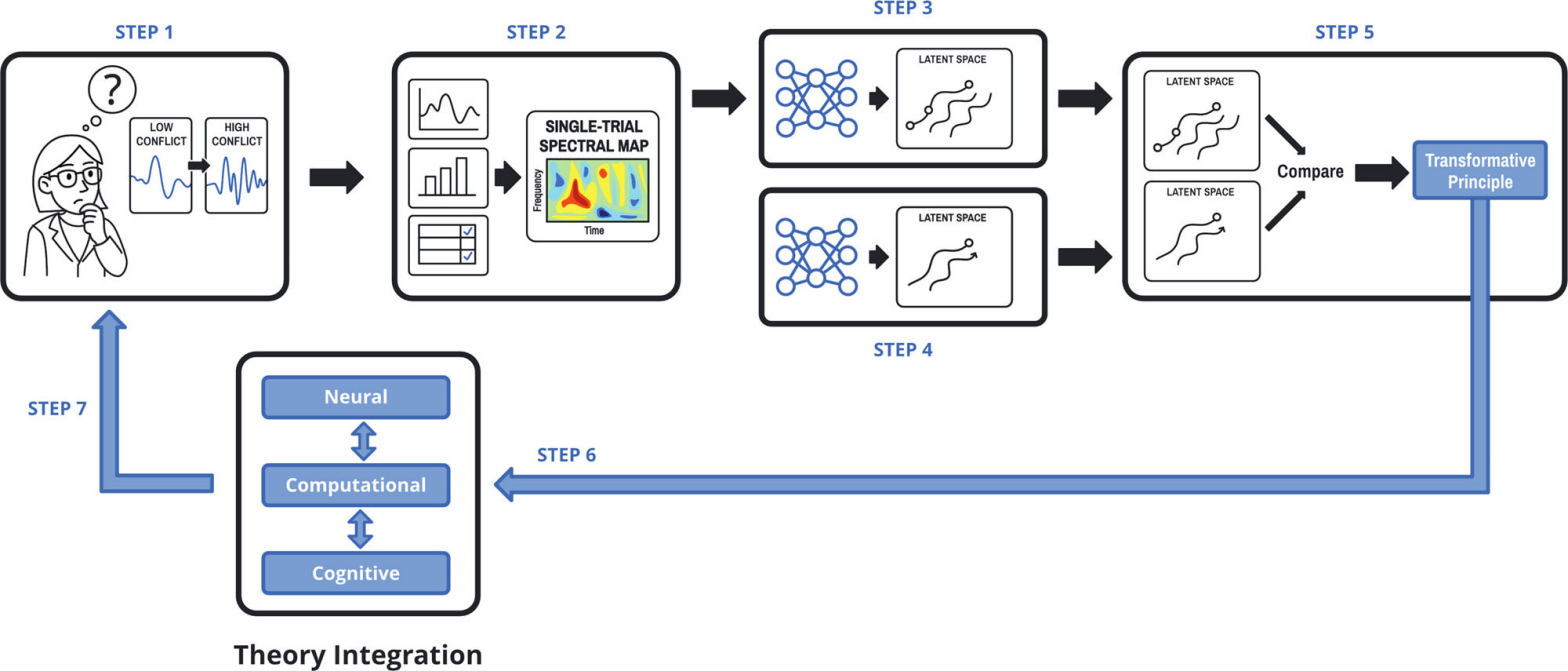
Generative-model workflow for relational neural analysis. The figure outlines a six-step pipeline: Step 1: formulating a relational hypothesis (e.g., transforming low- to high-conflict neural states). This step defines the cognitive manipulation of interest and frames the central question. Step 2: preparing trial-wise EEG features as single-trial spectral maps. This preserves trial-by-trial variability and provides a rich, high-dimensional description of neural activity. Step 3: training generative models to learn latent representations for each condition. Single-trial spectral maps are transformed into a low-dimensional latent space using a multivariate or generative mapping (e.g., neural network-based embedding). The resulting latent trajectories represent the evolution of neural population states within a compact, geometry-based representation. Step 4: latent representations are derived separately for different cognitive processes (e.g., high vs. low conflict). This yields condition-specific trajectories within latent space, allowing direct comparison of how neural dynamics unfold under distinct task demands. Differences in trajectory shape, direction, or dispersion reflect systematic reconfiguration of oscillatory dynamics as a function of control requirements. Step 5: comparing latent-space trajectories to extract the underlying transformative principle. This step evaluates whether the transformations between latent spaces differ across conditions, thereby isolating the transformative principle linking cognitive demand to neural dynamics. Step 6: the identified transformative principle is interpreted across neural, computational, and cognitive levels. This step links latent-space geometry to computational mechanisms (e.g., state transitions, control allocation) and embeds them within a broader cognitive theory framework. Step 7: feeding these insights back into neural, computational, and cognitive theory integration. This guides the design of follow-up experiments, enabling an iterative cycle in which theoretical predictions are tested and refined.

By reverse engineering and inspecting a model’s latent space and transformation functions, researchers can access the geometry transformation that the model learned^88,89^. This can answer questions about whether and how two states differ along a single continuous axis or multiple orthogonal ones. Such questions are common across many fields of cognitive neuroscience, such as action control^74,90,91^ and perception^92,93^. Generative AI provides a computational key that helps cognitive neuroscience infer the principles underlying its transformations, enabling direct testing of long-standing questions, such as: (i) whether seemingly distinct mental operations occupy separate regions of neural state space, or whether they are continuous variations within a shared representational manifold (ii) whether perception, memory, emotion, and action interact through distinct modules or as transformations of a basic overarching computation (iii) whether neural states underlying different tasks, contexts, or modalities be aligned through learned mappings, revealing underlying common principles of computation (iv) whether the brain’s representational geometry reorganizes across time, during learning, development, or adaptation to new demands (but also see section on “Caveats of using generative AI for cognitive neuroscience”). Figure 2.

## Quantifying the success and failure of generative AI

The key to applying generative AI models to brain data is to determine whether a mapping between two neural states has been successful and, ultimately, conceptually meaningful. A successful mapping means that the generative model has learned a transformation that reproduces the structure and function of the target neural data. Success must therefore be defined in multidimensional terms. In principle, several routes are available to do so, which refer to (i) reconstruction fidelity^94^, (ii) preservation of representational geometry^95^, and (iii) functional equivalence^96^.

Reconstruction fidelity, as a first indicator of success or failure, is the extent to which the generated neural patterns reproduce the measured empirical data. This can be evaluated by comparing predicted and observed activity patterns, for example, through measures of overall similarity or reconstruction error. One may also compute the correlation between observed and predicted signals or the difference in activation patterns across all electrodes, sensors, or voxels. Statistical significance can be assessed with conventional methods, such as permutation or bootstrap tests, as well as Bayesian statistics^72,97^. The latter provides a direct quantification for a lack of difference and thus the success of a generative mapping. When models output probability distributions, their performance can be quantified by how well these distributions match those of the actual data. High fidelity then indicates that the model captures the relevant variance in the target condition.

However, beyond point-by-point similarity, a generative mapping should preserve the relational structure of the data; that is, the shape of the neural manifold. The preservation of the geometry can be tested by comparing the pattern of distances among all conditions or time points in the mapped data to those in the actual data. If the relative distances and neighborhood relations are preserved, the model has maintained the representational geometry. Techniques such as Procrustes alignment or subspace-overlap measures can be used to quantify this preservation^98^.

Yet, a mapping may be geometrically accurate but functionally meaningless. To test whether the transformed data (i.e., the synthetic neural activity generated by the model) retain task-relevant information and show functional equivalence (i.e., the ability of the generated data to decode task states or predict behavioral outcomes just as accurately as the real empirical data)^99^, one can apply classifiers or regression models trained on the real target data to the newly generated data^100,101^. If performance remains comparable, the mapping has preserved the functional code that links neural states to behavior or task variables. Failure in any of these three domains indicates a partial or unsuccessful transformation.

## A roadmap for applying generative AI to cognitive neuroscience research

The task ahead is to transform abundant neural data into conceptual insight. At an abstract level, the currently applied relational emphasis bears some resemblance to older associationism traditions, in that cognition is not treated as a set of isolated contents. However, the present framework differs in a fundamental respect: it formalizes relations as empirically testable transformations in neural and latent computational spaces, rather than as descriptive laws governing the succession of ideas. The generative AI modeling approach discussed may offer a path to do so since generative modelling is treated as a bridge serving various needs: (i) from data to theory, by revealing geometric structure that constrains hypotheses about how cognitive states relate, separate, and transform; (ii) from empirical patterns to (putative) mechanisms, by linking learned transformations to models of neural dynamics; and (iii) from isolated experimental paradigms to integrated architectures, by mapping how supposedly different cognitive functions and underlying computations connect^102^. Several steps seem necessary: first, theoretical propositions are reformulated as relational hypotheses, asking whether neural state A can be transformed into neural state B. Second, suitable datasets (i.e., large, structured, and cognitively annotated) are to be identified to train generative models. Third, the representational geometry of the latent space is quantified to reveal how cognitive states are arranged. Fourth, the mathematical principles of transformation are extracted using sensitivity, manifold, or information-theoretic analyses. Fifth, these principles are tested for generality across tasks, modalities, and populations, providing a basis for evaluating shared versus distinct cognitive mechanisms. Finally, the findings are integrated into a broader cognitive theory: successful transformations provide evidence compatible with shared representational structure, whereas failures suggest that the tested states may occupy separable geometries under the model class, measurement modality, and validation criteria used. While the final validation of such models typically relies on correlating generated outputs with empirical observations, the constraint imposed by out-of-distribution condition transfer provides a method to interrogate the robustness and generality of the underlying computational rules. This contrasts with traditional approaches that primarily identify differences without explicitly modeling generative alignment. Overall, this roadmap translates generative modeling into a practical logic of inquiry applicable across various research programs and will have an impact in the field of cognitive neuroscience (see Box 2).

Box 2: why a generative–transformational logic matters for cognitive neuroscience *It reveals relational structure*, not only differences. Generative models test whether cognitive states lie on a shared manifold or reflect separable mechanisms.

*It makes counterfactuals empirically tractable*. Generative mappings allow systematic simulation of how neural states would look under unobserved conditions (up to a limit).

*It connects machine-learned transformations with neurocomputational theory*. Learned mappings can be compared to attractor dynamics, predictive coding, or mixed-selectivity architectures.

*It provides mechanistic insight into clinical and developmental conditions*. Failure to transform between populations (e.g., patients vs. controls) implies distortions in representational geometry.

## Caveats of using generative AI for cognitive neuroscience

Despite the enormous potential of generative AI for cognitive neuroscience, there are also potential caveats to consider. First, generative AI models often require extremely large, labeled datasets to learn insightful patterns. Although there is a growing body of open datasets^5,103–108^ in neuroscience, considerable heterogeneity still remains. Furthermore, in many real contexts, large-scale datasets are hard to collect, for instance, due to limited sample sizes and the complexity of neural data collection^109,110^. Moreover, brain data is also highly variable across individuals and experimental conditions, making it challenging to gather the consistent, large-scale corpora needed for training generative AI models. “Second, generative AI models, despite their remarkable success, are often considered black boxes.”^111^ One reason frequently noted is that the internal decision-making processes of generative AI models are difficult to interpret, even when they produce accurate outputs. In cognitive neuroscience, this lack of transparency could limit understanding of the underlying neural substrates. Along the same line, in some generative AI models, for instance, in variational autoencoders, it is crucial to note that they do not yield interpretable latent dimensions by default (interpretability is not an inherent property of this model class), rather, it depends critically on the structure of the training data, the experimental design, specific inductive biases imposed on the model (such as disentanglement constraints), and careful post hoc analytic choices. Thus, in domains where mechanistic insight is essential (e.g., human pharmacology and brain stimulation), mere prediction based generative AI model is not sufficient, and a detailed understanding of the underlying physiological process is essential (which may not be attainable with biology-agnostic generative AI models). Thus, future endeavors to incorporate generative AI into cognitive neuroscience should also be mindful of these potential limitations.

A further caveat concerns the term “generative.” Some neural-state translation architectures operate deterministically on a fixed input vector. Such models may still be useful as learned mappings or transport functions between neural state spaces, but they should not be interpreted as modeling the full conditional distribution unless they include an effective stochastic sampling mechanism. Thus, when trial-level variability is theoretically important, models should be evaluated not only by pointwise reconstruction accuracy, but also by whether repeated samples reproduce the variance, covariance structure, and task-relevant distributional properties of the empirical target state.

Lastly, skeptical takes^112^ highlight that even when generative models produce highly accurate data transformations, we must rigorously question whether the model’s internal representational geometry is isomorphic with the brain’s internal representational structure. Mere matching outputs does not guarantee that the model has captured the biologically correct generative process. Critics argue that these models may be discovering highly sophisticated “shortcuts” or descriptive statistical mappings that are effective but are conceptually misaligned with the actual mechanistic operations performed by neuronal circuits. This critique reinforces that the latent spaces discovered by generative AI should be treated as hypothesis-generating tools whose representations require independent, orthogonal neurophysiological validation, rather than being accepted as definitive mechanistic explanations on their own.

## Summary

Generative modeling extends the geometric turn in cognitive neuroscience by shifting the focus from describing neural states to modeling the transformations that link them. It provides a way to test whether cognitive operations might share a common representational geometry or rely on distinct neural mechanisms. By quantifying the success and failure of such transformations, theoretical distinctions become empirically tractable. This generative framework helps cognitive neuroscience move from mapping correlates toward testing candidate mechanistic relationships among neural states. Ultimately, it better connects cognitive theory with neural data^113^.

### Reporting summary

Further information on research design is available in the Nature Portfolio Reporting Summary linked to this article.

## Supplementary information

Reporting Summary
